# Numerical study of a confined slot impinging jet with nanofluids

**DOI:** 10.1186/1556-276X-6-188

**Published:** 2011-03-01

**Authors:** Oronzio Manca, Paolo Mesolella, Sergio Nardini, Daniele Ricci

**Affiliations:** 1 Dipartimento di Ingegneria Aerospaziale e Meccanica, Seconda Università degli Studi di Napoli, Via Roma 29 - 81031 Aversa, Italy

## Abstract

**Background:**

Heat transfer enhancement technology concerns with the aim of developing more efficient systems to satisfy the increasing demands of many applications in the fields of automotive, aerospace, electronic and process industry. A solution for obtaining efficient cooling systems is represented by the use of confined or unconfined impinging jets. Moreover, the possibility of increasing the thermal performances of the working fluids can be taken into account, and the introduction of nanoparticles in a base fluid can be considered.

**Results:**

In this article, a numerical investigation on confined impinging slot jet working with a mixture of water and Al_2_O_3 _nanoparticles is described. The flow is turbulent and a constant temperature is applied on the impinging. A single-phase model approach has been adopted. Different geometric ratios, particle volume concentrations and Reynolds number have been considered to study the behavior of the system in terms of average and local Nusselt number, convective heat transfer coefficient and required pumping power profiles, temperature fields and stream function contours.

**Conclusions:**

The dimensionless stream function contours show that the intensity and size of the vortex structures depend on the confining effects, given by *H/*W ratio, Reynolds number and particle concentrations. Furthermore, for increasing concentrations, nanofluids realize increasing fluid bulk temperature, as a result of the elevated thermal conductivity of mixtures. The local Nusselt number profiles show the highest values at the stagnation point, and the lowest at the end of the heated plate. The average Nusselt number increases for increasing particle concentrations and Reynolds numbers; moreover, the highest values are observed for *H/W *= 10, and a maximum increase of 18% is detected at a concentration equal to 6%. The required pumping power as well as Reynolds number increases and particle concentrations grow, which is almost 4.8 times greater than the values calculated in the case of base fluid.

List of symbols

## Background

Heat transfer enhancement is very important in the industry, and several techniques are employed to realize this aim. Impinging jets, whether confined or unconfined, have been widely used for efficient cooling in industrial applications as a means of providing highly localized heat transfer coefficients, representing a possible solution. Depending on the application, flow conditions can range from laminar to highly turbulent ones. Applications of impinging jets include drying of textiles, film and paper, cooling of gas turbine components and the outer walls of combustors, freezing of tissue in cryosurgery and manufacturing, material processing and electronic cooling. There are numerous articles dealing with this problem both numerically and experimentally as reported in the literature reviews on the subject [1-6].

Several studies have been developed on impinging air jets [1,2]. Recently, a greater attention has been dedicated to the impinging liquid jet since orders of magnitude of the heat transfer rates are several times those of gas jets. Liquid jets have possible application to the cooling of heat engines [5,7], thermal control in electronic devices [8-10] and in the thermal treatment of metals and material processing [11-14].

In the application of jet impingements, circular or slot jets are the main jet configurations. For these two configurations, flow and heat transfer mechanics are significantly different. It seems that greater research activity on heat and mass transfer with circular impinging jets has been predominantly published [1-3,15,16]. However, investigations on heat and mass transfer with slot jet impingement have attracted more attention recently. In fact, slot jet impingements offer many more beneficial features, such as higher cooling effectiveness, greater uniformity and more controllability, as underlined in [17]. For example, these factors allow for fulfillment of the increasing heat flux and decreasing dimensions in electronics packages [17-24]. The common types of impinging jets are with or without confinement. Confined impinging jets have the advantages of smaller space design, while unconfined impinging jets have an advantage of simple design and easy fabrication. The two types of impinging jets have their own merits, and they are both commonly used as the cooling solutions, and the literature reviews on the subject have been provided in [2,3,6]. The effects of confinement on impinging jet heat transfer have been considered in [25-27]. Moreover, several studies show the importance of the subject and different cases have been investigated, such as confined slot-jet impingement on a moving plate [28], impinging jet on obliquely a flat surface [29], impinging jet on a porous medium [30] and slot jet impingement cooling on a semi-circular concave [31].

In order to obtain a heat transfer enhancement in jet impingement, different techniques have been employed, such as the insert of foams or fins [32]. These techniques determine a modification of the cooling system whereas the use of nanofluids in a coolant seems to be simpler in realizing a heat transfer enhancement [33]. However, nanofluids are to this day controversial in many areas such as inconsistencies in published data and disagreements on the heat transfer mechanisms, as observed by Gherasim et al. [34]. Various aspects of nanofluids have been covered in several reviews and some of these are given in [35-47].

The employment of nanofluids in impinging jets has been investigated recently by some researchers and, to the best of our knowledge, their investigations have been reported in [34,48-60]. The numerical investigation on hydrodynamic and thermal fields of Al_2_O_3_/water nanofluid in a radial laminar flow cooling system carried out by Roy et al. [48] can be can be considered as the first article on an impinging jet. Those authors found that considerable heat transfer enhancement was observed up to 200% in the case of a nanofluid with 10% in nanoparticle volume concentration at a Reynolds number equal to 1200. However, a significant increase in wall shear stress was noticed increasing the nanoparticle volume concentration. The laminar-forced convection flow of nanofluids between two coaxial and parallel disks with central axial injection was investigated numerically considering temperature-dependent properties by Palm et al. [51]. Results indicated a heat transfer benefit by adopting Al_2_O_3_/water nanofluid with a volume fraction of nanoparticles of 4%. An increase of 25% was evaluated in terms of average wall heat transfer coefficient, when referred to the water. Moreover, the use of temperature-dependent properties determined for greater heat transfer predictions with corresponding decreases in wall shear stresses when compared to evaluations employing constant properties. A numerical study on steady, laminar radial flow of a nanofluid in a simplified axi-symmetric configuration with axial coolant injection was performed by Roy et al. [52] for electronic cooling applications. Also in this investigation increases in heat removal capabilities were detected with the use of nanofluids.

An experimental investigation in a confined and submerged impinging jet on a flat, horizontal and circular heated surface with nanofluid (Al_2_O_3 _dispersed in water) was carried out by Nguyen et al. [56]. Experimental results were obtained for both laminar and turbulent flow regimes and they showed that, depending on the combination of nozzle-to-heated surface distance and particle volume fraction, the use of a nanofluid can determine a heat transfer enhancement in some cases, but an adverse effect on the convective heat transfer coefficient may occur in other cases. A circular confined and submerged jet impinging on a horizontal hot plate was numerically simulated by Vaziei and Abouali [57]. Water and 36-nm Al_2_O_3_-water nanofluid with various particle volume fractions were considered as a working fluid for cooling the hot plate. Both laminar and turbulent impinging jets in various nozzle-to-plate distances and Reynolds numbers were simulated. The results showed that the use of Al_2_O_3 _nanoparticles in laminar jets enhanced the heat transfer but for the turbulent jets Al_2_O_3_-water nanofluid had a lower performance for heat removal compared with the base fluid. The heat transfer enhancement capabilities of Al_2_O_3_/water inside a confined impinging jet cooling device was numerically studied by Gherasim et al. [34]. Results highlighted those limitations in the use of this nanofluid type in a radial flow configuration, due to the significant increase in the associated pumping power. Steady laminar incompressible thermal alumina-water flow between parallel disks was simulated by Feng and Kleinstreuer [58]. The results indicated that the Nusselt number increases with higher nanoparticle volume fraction, smaller nanoparticle diameter, reduced disk-spacing and larger inlet Reynolds number. The laminar forced convective heat transfer features of Al_2_O_3_/water nanofluid in the confined radial flow were numerically investigated by Yang and Lai [59,60] with constant [59] and temperature-dependent properties [60]. Results showed the same trend given in the previous published works: the Nusselt number increases with the increases in Reynolds number and nanoparticle volume fraction, though the increase in pressure drop is more significant with the increase of particle concentration. Furthermore, temperature-dependent thermo-physical properties of nanofluids were found to have a marked bearing on the simulation results.

It seems that a slot-confined and submerged impinging jet on a flat surface with nanofluids has not been investigated in both laminar and turbulent flow regimes in spite of its importance in engineering applications such as electronic cooling and material processing.

In this article, a numerical investigation on turbulent flow on a slot-confined and submerged impinging jet on an isothermal flat surface is carried out. The results are given to evaluate the fluid dynamic and thermal features of the considered geometry with Al_2_O_3_/water as the working nanofluid adopting the single phase model.

## Methods

### Geometrical model

**Figure 1:**
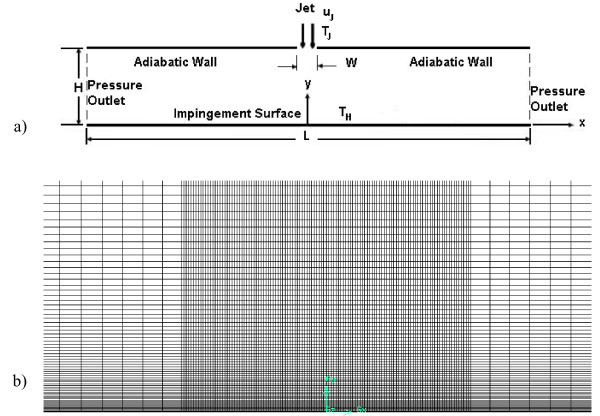
**Sketch of the model**. **(a) **Geometry and boundary conditions; **(b) **details on the adopted mesh near the impingement surface.

A computational thermo-fluid dynamic analysis of a two-dimensional model, Figure 1a, which regards the impinging jet on a heated wall with nanofluids, is considered in order to evaluate the thermal and fluid-dynamic performances, and study the velocity and temperature fields. The two-dimensional model has a length *L *equal to 310 mm while the height *H *ranges from 24.8 to 124 mm and the jet orifice width *W *is 6.2 mm. A constant temperature value of 343 K is applied on the impingement bottom surface. Different values of *H/W *ratio, equal to 4, 6, 8, 10, 15 and 20, are considered. The working fluid is water or a mixture of water and *γ*-Al_2_O_3 _nanoparticles with a diameter of 38 nm, at different volume fractions equal to 1, 4 and 6%.

### Physical properties of nanofluids

The working fluid is water or a mixture of water and *γ*-Al_2_O_3 _nanoparticles with a diameter of 38 nm, at different volume fractions equal to 1, 4 and 6%. In Table 1, the values of density, specific heat, dynamic viscosity and thermal conductivity, given by Rohsenow et al. [61], are reported for water and *γ*-Al_2_O_3_. The presence of nanoparticles and their concentrations influence the mixture properties. A single-phase model was adopted, and the following equations were used for computing the thermal and physical properties of the considered nanofluids [62-65], given in Table 2. Density was evaluated using the classical formula developed for conventional solid-liquid mixtures, while the specific heat values were obtained by assuming the thermal equilibrium between particles and surrounding fluid [62,63].

**Table 1:** Material properties at a temperature of 293 K

Material	***ρ *(kg/m**^**3**^**)**	***c***_***p ***_**(J/kg K)**	*μ *(Pa s)	*k *(W/m K)
*γ*-Alumina (Al_2_O_3_)	3880	773	//	36
Water	998	4182	998 × 10^-6^	0.597

**Table 2:** Properties of nanofluids, *single-phase model*.

*ϕ*	***ρ *(kg/m**^**3**^**)**	***c***_***p ***_**(J/kg K)**	*μ *(Pa s)	*k *(W/m K)
0%	998.2	4182	998 × 10^-6^	0.597
1%	1027	4148	1083 × 10^-6^	0.614
4%	1113	4046	1486 × 10^-6^	0.667
6%	1171	3977	1877 × 10^-6^	0.705

(1) $$\text{Density: }\rho_{\text{nf}}=(1-\phi )\rho_{\text{bf}}+\phi \rho_{\text{p}}$$

(2) $$\text{Specific heat: }c_{p_{\text{nf}}}=(1-\phi )c_{p_{\text{bf}}}+\phi c_{p_{\text{p}}}$$

Nanofluids may be considered as Newtonian fluids for low volume fractions, e.g., up to 10%, and for small temperature increases. In this way, for the viscosity as well as for thermal conductivity, formulas given by [64,65] were adopted:

(3) $$\text{Dynamic viscosity: }\mu_{\text{nf}}=\mu_{\text{bf}}(123\phi^{2}+7.3\phi +1)$$

(4) $$\text{Thermal conductivity: }k_{\text{nf}}=k_{\text{bf}}(4.97\phi^{2}+2.72\phi +1)$$

However, it is well known that the evaluation of these properties by various research groups differs from each other because of the numerical and experimental approaches and processes adopted [64,65].

### Mathematical description and governing equations

Steady-state, turbulent, incompressible, single-phase, and constant properties flow conditions are considered in the present analysis. The governing equations of continuity, momentum and energy are solved in rectangular coordinates:

(5) $$\text{Continuity: }\frac{\partial }{\partial x_{i}}\left(\rho u_{i}\right)=0$$

(6) $$\text{Mom}\text{.: }\frac{\partial }{\partial x_{j}}\left(\rho u_{i}u_{j}\right)=-\frac{\partial P}{\partial x_{i}}+\frac{\partial }{\partial x_{j}}\left[\mu \left(\frac{\partial u_{i}}{\partial x_{j}}+\frac{\partial u_{j}}{\partial x_{i}}-\frac{2}{3}\delta_{ij}\frac{\partial u_{i}}{\partial x_{j}}\right)\right]+\frac{\partial }{\partial x_{j}}\left(-\rho \overset{\_\_\_\_\_\_\_\_}{{u^{\prime }}_{i}{u^{\prime }}_{j}}\right)$$

(7) $$\text{Energy: }\frac{\partial }{\partial x_{i}}\left[u_{i}\left(\rho E+P\right)\right]=\frac{\partial }{\partial x_{j}}\left[\left(\lambda +\frac{c_{p}\mu_{\text{t}}}{Pr_{\text{t}}}\right)\frac{\partial T}{\partial x_{j}}+u_{i}{\left(\tau_{ij}\right)}_{\text{eff}}\right]$$

where *E *is the total energy, $E=c_{p}T-\frac{P}{\rho }+\frac{u^{2}}{2}$ and (*τ*_ij_)_eff _is the deviatoric stress tensor, defined as

(8) $${\left(\tau_{ij}\right)}_{\text{eff}}=\mu_{\text{eff}}\left(\frac{\partial u_{j}}{\partial x_{i}}+\frac{\partial u_{i}}{\partial x_{j}}\right)-\frac{2}{3}\mu_{\text{eff}}\frac{\partial u_{i}}{\partial x_{j}}\delta_{ij}$$

The *k-ε standard *model with *enhanced wall treatment *is assumed. The transport equations are as follows [66]:

(9) $$\frac{\partial }{\partial t}\left(\rho k\right)+\frac{\partial }{\partial x_{i}}\left(\rho ku_{i}\right)=\left[\frac{\partial }{\partial x_{j}}\left(\mu +\frac{\mu_{t}}{\sigma_{k}}\right)\frac{\partial k}{\partial x_{i}}\right]+\left(G_{\text{k}}+G_{\text{b}}\right)-\rho \varepsilon -Y_{M}+S_{\varepsilon }$$

(10) $$\frac{\partial }{\partial t}\left(\rho \varepsilon \right)+\frac{\partial }{\partial x_{i}}\left(\rho \varepsilon u_{i}\right)=\left[\frac{\partial }{\partial x_{j}}\left(\mu +\frac{\mu_{t}}{\sigma_{\varepsilon }}\right)\frac{\partial \varepsilon }{\partial x_{i}}\right]+C_{1}\frac{\varepsilon }{k}\left(G_{\text{k}}+C_{3\varepsilon }G_{\text{b}}\right)-C_{2\varepsilon }\rho \frac{\varepsilon^{2}}{k}+S_{\varepsilon }$$

where *G*_k _is the production of turbulent kinetic energy due to mean velocity gradients, *G*_b _represents the generation of the turbulent kinetic energy due to buoyancy while *Y*_*M *_is referred to the fluctuation rates related to the overall dissipated turbulent thermal energy. In particular, *G*_k _may be expressed by

(11) $$G_{\text{k}}=-\rho \bar{{u^{\prime }}_{i}{u^{\prime }}_{j}}\frac{\partial u_{j}}{\partial x_{i}}$$

where *C*_1__*ε *_and *C*_2__*ε *_are constants; while the term $C_{3\varepsilon }=\tanh\left|\frac{v}{u}\right|$ defines the dependence rate of *ε *on buoyancy; *σ*_*k *_and *σ*_*e *_represent the turbulent Prandtl numbers based on *k *and *ε*, respectively; while *S*_*k *_and *S*_*ε *_are further generation terms. The turbulent viscosity is defined by

(12) $$\mu_{\text{t}}=\rho C_{\mu }\frac{k^{2}}{\varepsilon }$$

where *C*_*μ *_is a constant. The model constant values are the following:

*C*_1*ε *_= 1.44, *C*_2*ε *_= 1.92, *C*_*μ *_= 0.09, *σ*_k _= 1.0 and *σ*_*ε *_= 1.3.

The *enhanced wall treatment *approach has been considered. The assigned boundary conditions are

- Inlet jet section: uniform velocity and temperature profile;

- Outlet section: pressure outlet;

- Bottom wall: velocity components equal to zero and constant temperature;

- Upper wall: velocity components equal to zero and adiabatic condition.

The dimensionless parameters considered here are

(13) $$Re=\frac{u_{\text{J}}W}{\nu }$$

(14) $$Nu=\frac{\dot{q}W}{\left(T_{\text{H}}-T_{\text{J}}\right)\lambda_{\text{f}}}$$

(15) $$\theta =\frac{T-T_{J}}{\left(T_{H}-T_{J}\right)}$$

where *u*_*j *_is the jet velocity, *W *is the jet width, $\dot{q}$ is the impingement surface heat flux, *T*_H _and *T*_J _represent the temperature of the impingement surface and the jet temperature, respectively.

### Numerical procedure

The governing equations of continuity, momentum and energy, reported in the previous section, are solved by the finite volume method by means of FLUENT code [67]. A steady-state solution and a segregated method are chosen to solve the governing equations, which are linearized implicitly with respect to dependent variables of the equation. A second-order upwind scheme is chosen for energy and momentum equations. The SIMPLE coupling is chosen as scheme to couple pressure and velocity. The convergence criteria of 10^-5 ^for the residuals of the velocity components and of 10^-8 ^for the residuals of the energy are assumed. It is assumed that the incoming flow is turbulent at ambient temperature and pressure. Different inlet uniform velocities, corresponding to Reynolds numbers ranging from 5000 to 20000, were considered and they are reported in Table 3. Furthermore, the inlet turbulence intensity value is set to 2%.

**Table 3:** Inlet velocities (m/s).

*Re*	Water	Water/alumina 1%	Water/alumina 4%	Water/alumina 6%
5000	0.81	0.85	1.08	1.29
10000	1.61	1.70	2.15	2.59
15000	2.42	2.55	3.23	3.88
20000	3.23	3.40	4.30	5.17

The *enhanced wall treatment *functions are activated to increase the model accuracy in the near-wall region. It is a *two-layer *method with enhanced functions. The domain is divided into two regions, the near-wall region and the core ones, according to the turbulent Reynolds number, based on the distance-to-wall term *y*.

(16) $$Re_{y}=\frac{\rho y\sqrt{k}}{\mu }$$

The core region, for *Re*_*y *_> 200, is solved by means of the *standard k-ε model*, while in the other region the Wolfstein model is applied [68].

Along the solid walls, no slip condition is employed, whereas a velocity inlet is given for the jet orifice and pressure conditions are set for the outlet sections.

Four different grid distributions are tested on the model with *H/W *ratio equal to 6 at *Re *= 5000, with water (*ϕ *= 0%) as working fluid, to ensure that the calculated results are grid independent. The four grids have 4950 (90 × 55), 19800 (180 × 110), 79200 (360 × 220), and 316800 (720 × 440) nodes, respectively. The grid mesh is structured in each case with grid adoption for *y*^+ ^= 1 at adjacent wall region and a sketch is shown in Figure 1b. For the adiabatic wall and the bottom surface, nodes are distributed by means of an exponential relation (*n *= 0.9), to have a fine mesh near the impingement region, where an equi-spatial distribution is chosen. On the vertical ones, a bi-exponential (*n *= 0.8) distribution is considered.

**Figure 2:**
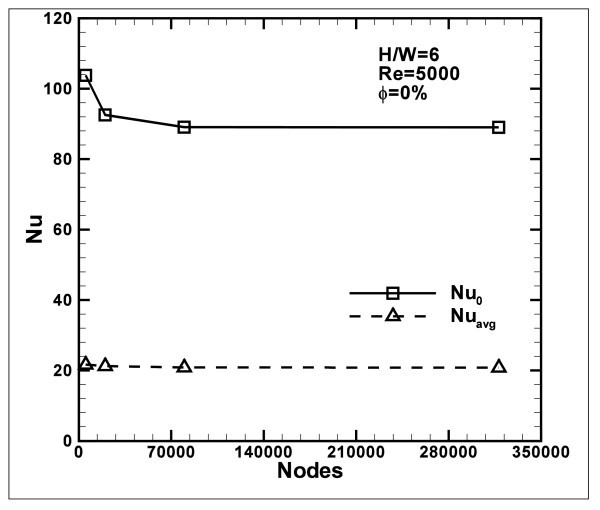
**Analysis of grid independence in terms of Nusselt number, *H/W *= 6, *R *= 5000 and ***ϕ***= 0%**.

Comparing the third- and fourth-mesh configurations, in terms of average and stagnation point Nusselt number, results are very close, and the relative errors are very little, as reported in Figure 2. As a result, the third grid case has been adopted because it ensured a good compatibility between the machine computational time and the accuracy requirements.

**Figure 3:**
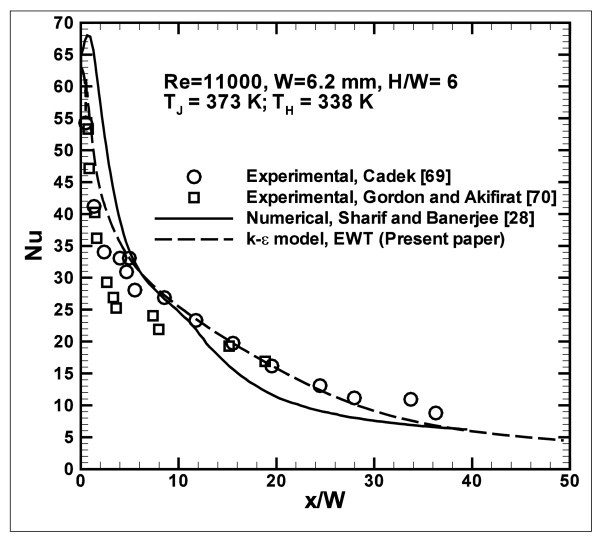
**Validation of numerical results in terms of Nusselt number**.

Results are validated by comparing the obtained numerical data with the experimental and numerical ones, given in [28,69,70]. Figure 3 presents the comparison in terms of average Nusselt number profiles, for the cases, characterized by *Re *= 11000, *H/W *= 6, *T*_J _= 373 K and *T*_H _= 338 K. It is observed that the numerical results, obtained in this work, fit very well with the experimental ones given in [5,6] both near the stagnation point region and at the side one.

## Results and discussion

A computational thermo-fluid dynamic analysis of a two-dimensional model, regarding a confined impinging jet on a heated wall with nanofluids, is considered to evaluate the thermal and fluid-dynamic performances and study the velocity and temperature fields. Different inlet velocities are considered to ensure a turbulent regime, and the working fluids are water and mixtures of water and *γ*-Al_2_O_3 _at different volume fractions, treated by a single-phase model approach. The range of Reynolds numbers, geometric ratio and volume fractions are given below:

• Reynolds number, *Re*: 5000, 10000, 15000 and 20000;

• *H/W *ratio: 4, 6, 8, 10, 15 and 20;

• particle concentrations, *ϕ*: 0, 1, 4 and 6%.

Results are presented in terms of average and local Nusselt number profiles, as a function of Reynolds number, *H/W *ratio and particle concentrations; moreover, dimensionless temperature fields and stream function contours are provided.

**Figure 4:**
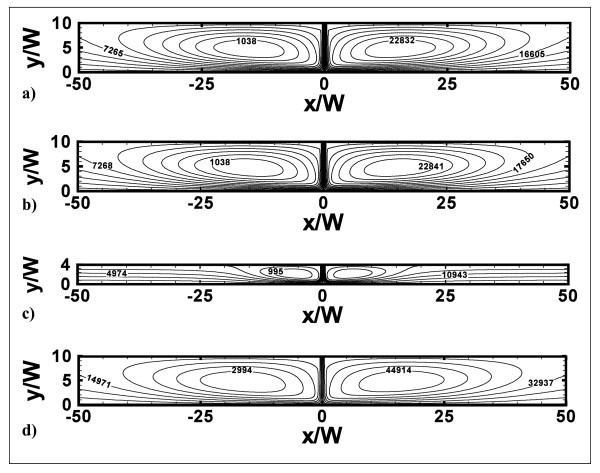
**Stream functions contours**. **(a) ***H/W *= 10, *Re *= 10000 and *ϕ *= 0%; **(b) ***H/W *= 10, *Re *= 10000 and *ϕ *= 4%; **(c) ***H/W *= 4, *Re *= 10000 and *ϕ *= 4%; **(d) ***H/W *= 10, *Re *= 20000 and *ϕ *= 4%.

**Figure 5:**
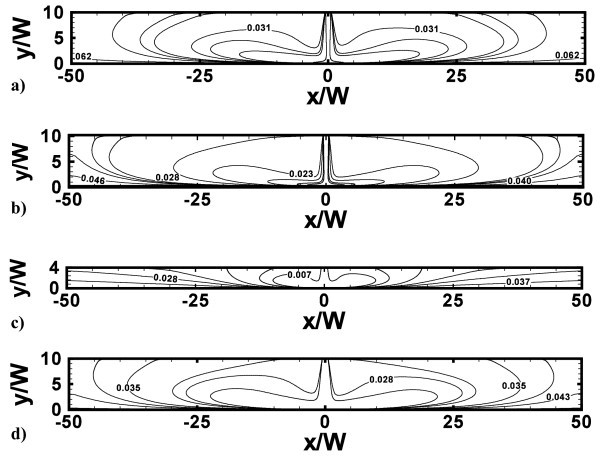
**Temperature fields**. **(a) ***H/W *= 10, *Re *= 10000 and *ϕ *= 0%; (b) *H/W *= 10, *Re *= 10000 and *ϕ *= 4%; **(c) ***H/W *= 4, *Re *= 10000 and *ϕ *= 4%; **(d) ***H/W *= 10, *Re *= 20000 and *ϕ *= 4%.

Figures 4 and 5 depict the stream lines contours and the temperature fields, respectively, for the representative cases with *H/W *= 4 and 10, at *Re *= 10000 and 20000 and *ϕ *= 0 and 4%. According to Figure 4, two counter-rotating vortex structures are generated as the jet impinges on the bottom surface and only one stagnation point, where velocity and temperature gradients are very high, is observed. This is due to the jet entrainment and confining effects of the upper adiabatic surfaces. Vortex intensity and size depend on *H/W *ratio, factors such as the confining effects, Reynolds number, and particle concentrations. It can be seen in Figure 4a, b, at *Re *= 10000, *H/W *= 10 and *ϕ *= 0 and 4%, the introduction of particles leads to a little smoother eddies with a low intensity increase, because the nanofluid viscosity is higher than water. As *H/W *ratio decreases from 10 to 4, at *Re *= 10000 and *ϕ *= 4%, vortices are less strong and smaller as they extinguish at *x/W *values equal to about -30 and 30, as pointed out in Figure 4b, c. As *Re *increases, the separation area near the inlet section becomes larger while the fluid stream results to be more compressed towards the impingement surface, as observed in Figure 4d.

The temperature fields, depicted in Figure 5, follow the stream line patterns. For increasing concentrations, nanoparticles produce an increase of fluid bulk temperature, because of the elevated thermal conductivity of mixtures. Near the impingement surface, temperature grows and tends to decrease for increasing *x/W *values. For larger Reynolds numbers, the efficiency of heat transfer increases.

**Figure 6:**
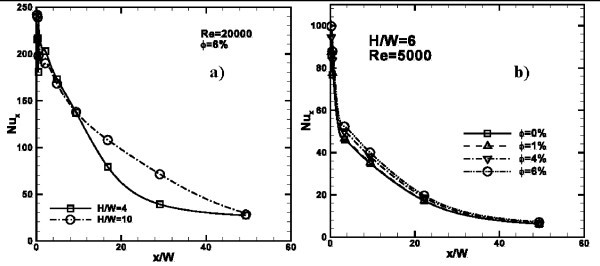
**Local Nusselt number profiles along *x/W***: **(a) ***H/W *= 4 and 10, *Re *= 20000, *ϕ *= 6%; **(b) ***H/W *= 6, *Re *= 5000, *ϕ *= 0, 1, 4 and 6%.

The variation of local Nusselt number along the impingement plate for *Re *= 20000, *H/W *= 4 and *ϕ *= 6% and for *Re *= 5000, *H/W *= 6 and different concentrations, is shown in Figure 6a, b, respectively. It is observed that the highest values of *Nu*_*x *_are evaluated at the stagnation point for all the considered cases; their values are 214 and 239 for *H/W *= 4 and *H/W *= 10, respectively. For low *H/W *values, local Nusselt number decreases more quickly than high *H/W *ratios. At the end of the plate, for any considered *H/W*, *Nu*_*x *_reaches similar values equal to about 25, as observed in Figure 6a. In Figure 6b, it is shown how the variation of nanofluid concentration affects the heat transfer. Higher heat transfer enhancements are observed for *ϕ *= 4, 6%, especially, near the impingement location. This does not happen only for *H/W *= 4 as can be understood from the average Nusselt number value trends, reported later, in comparison with other *H/W *ratios.

**Figure 7:**
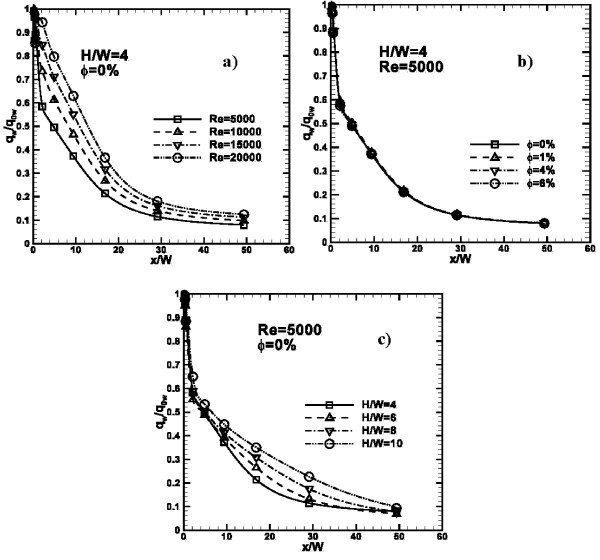
**Profiles of *q***_***w***_***/q***_***0w***_** ratio along *x/W***: **(a) ***H/W *= 4, *ϕ *= 0%, *Re *= 5000, 10000, 15000 and 20000; **(b) ***H/W *= 4, *Re *= 5000, *ϕ *= 0, 1, 4 and 6%; **(c) ***H/W *= 4, 6, 8 and 10, *ϕ *= 0% and *Re *= 5000.

In Figure 7, the variation of local *q*_*w*_*/q*_0__*w *_ratio is shown. The *q*_*w*_*/q*_0__*w *_value represents the local ratio between the local total heat flux and total heat flux at stagnation point for any case. The maximum value is reached at the stagnation point of any considered case. As *Re *increases, *q*_*w*_*/q*_0__*w *_ratio increases. Difference in terms of *q*_*w*_*/q*_0__*w *_is more significant passing from *Re *= 5000 to 10000 than the other considered *Re*. In fact, at *x/W *= 4, there is a difference of 0.12 in terms of *q*_*w*_*/q*_0__*w *_while in the other cases, the largest difference is 0.9. The heat transfer augmentation is more significant near the stagnation point than in correspondence with the end of the impinged plate. In Figure 7b, it is observed as the nanofluid concentration has very little influence on *q*_*w*_*/q*_0__*w*_. The effects of *H/W *are underlined in Figure 7c: near the stagnation point, *q*_*w*_*/q*_0__*w *_ratio has almost the same value for all *H/W*. From *x/W *= 4 curves spread out and *q*_*w*_*/q*_0__*w *_increases as *H/W *increases. This affects the results in terms of average Nusselt number, calculated at different *H/W *ratios.

**Figure 8:**
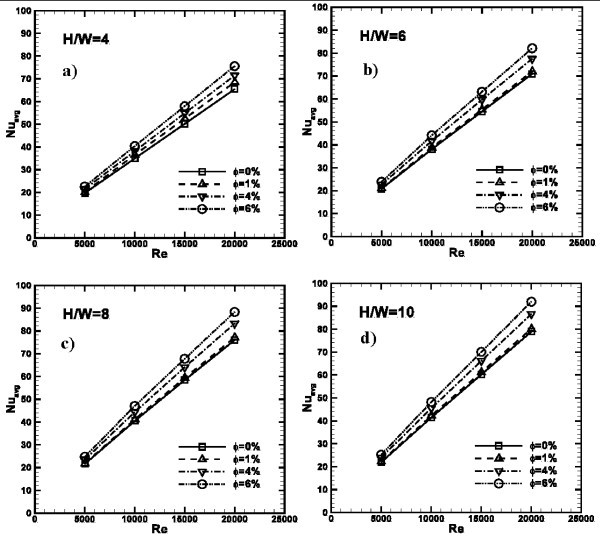
**Average Nusselt number profiles as function of *Re*, ***ϕ***= 0, 1, 4 and 6%**: **(a) ***H/W *= 4; **(b) ***H/W *= 6; **(c) ***H/W *= 8; **(d) ***H/W *= 10.

The average Nusselt number profiles as function of *Re *are depicted in Figure 8 for *H/W *= 4, 6, 8, and 10. Profiles increase as *Re *increases for all the considered cases. It is observed that as *ϕ *increases *Nu*_avg _becomes higher for a fixed value of *Re*. Passing from *ϕ *= 0% to *ϕ *= 1%, a significant increase of *Nu*_avg_, only for *H/W *= 4 configuration is noted, where it passes from 35 to 37 at *Re *= 15000 or 65 to 69 for *Re *= 20000. For the other cases, a significant heat transfer enhancement is found for the highest *ϕ *values; in fact in these cases, passing from *ϕ *= 0% to *ϕ *= 1%, the maximum enhancement is found to be equal to 1.22 times for *H/W *= 10 at *Re *= 20000.

**Figure 9:**
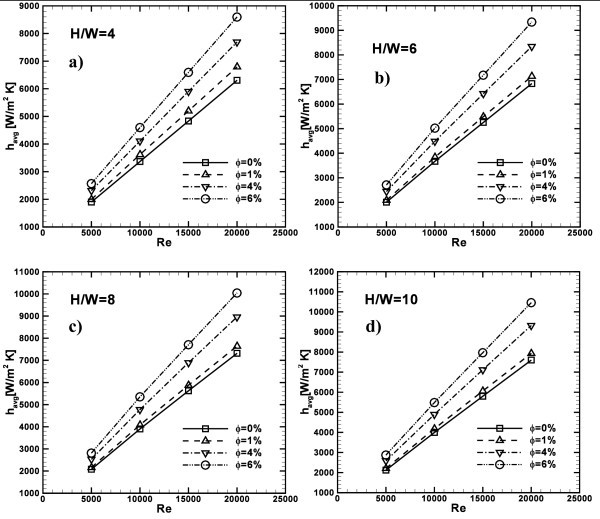
**Average convective heat transfer coefficient profiles as function of *Re*, ***ϕ***= 0, 1, 4 and 6%**: **(a) ***H/W *= 4; **(b) ***H/W *= 6; **(c) ***H/W *= 8; **(d) ***H/W *= 10.

The heat transfer enhancement is evident, also observing the average heat transfer coefficient profiles, described in Figure 9. Results are given for different *Re*, *H/W *ratios and concentrations. The maximum values of *h*_avg _are calculated for the highest values of *Re*, *H/W *and concentrations considered. In fact, for *H/W *= 10 and *Re *= 20000, it results that *h*_avg _is equal to about 7600, 8000, 9400 and 10500 W/m^2^K, as depicted in Figure 9d, while, at *H/W *= 4 and *Re *= 20000, *h*_avg _are equal to about 6200, 6800, 7700, and 8600 W/m^2^K, for *ϕ *= 0, 1, 4, and 6%, respectively.

**Figure 10:**
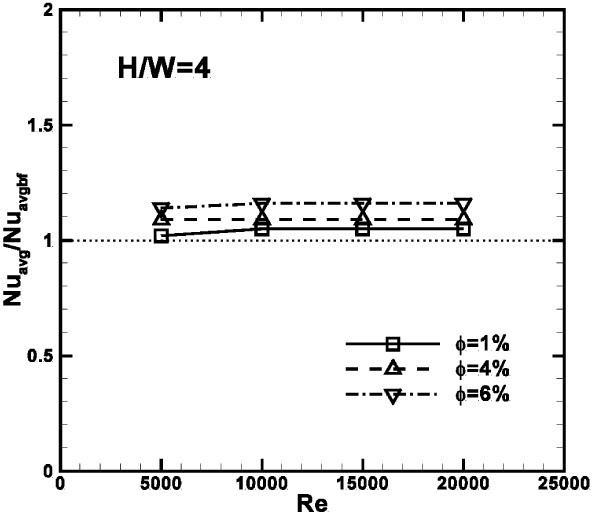
**Profiles of *Nu***_**avg**_***/Nu***_**avg, bf **_**ratio as a function of *Re *for different values of particle concentrations, *H/W *= 4**.

Figure 10 shows the average Nusselt number profiles, referred to the values calculated for the base fluid, as a function of Reynolds number for particle concentrations equal to 1, 4 and 6% at *H/W *ratio of 4. It is observed that the ratio *Nu*_avg_*/Nu*_avg, bf _is greater than one for all the configurations analyzed and rises slightly for increasing Reynolds numbers and concentrations; in fact, the highest value of 1.18 is detected at *Re *= 20000 and *ϕ *= 6%.

**Figure 11:**
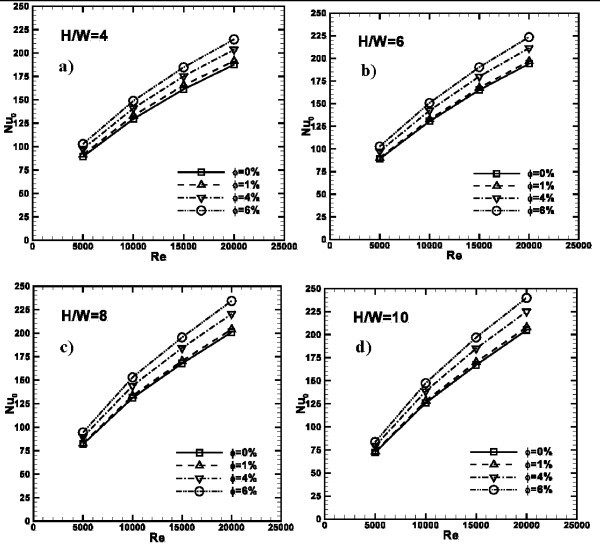
**Stagnation point values of local Nusselt number**. Values of local Nusselt number in correspondence with the stagnation point, for different *Re *and concentrations: **(a) ***H/W *= 4; **(b) ***H/W *= 6; **(c) ***H/W *= 8; **(d) ***H/W *= 10.

The results in terms of local Nusselt numbers, calculated for the stagnation point, are depicted in Figure 11. They are provided as a function of Reynolds numbers and given for different concentrations for different *H/W *ratios, equal to 4, 6, 8, and 10. It is shown that profiles increase almost linearly with increasing Reynolds numbers for all the considered concentrations and *H/W *ratios. Moreover, the *Nu*_*0 *_values are the highest for *ϕ *= 6% for all the considered Reynolds numbers. For example, comparing the results for *ϕ *= 1, 4, and 6%, with the base fluid ones, an increase in values of 2.7, 10.8, and 16.2% are detected for *H/W *= 4 at *Re *= 20000, respectively. Moreover, *Nu*_0 _values rises as *H/W *increases for *Re *> 10000, as observed in Figure 11b, c, d.

**Figure 12:**
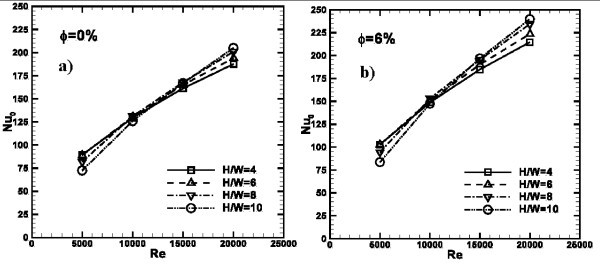
**Stagnation point Nusselt number values as a function of *Re***. Nusselt number values of stagnation point as a function of *Re*, for different *H/W *ratios: **(a) ***ϕ *= 0%; **(b) ***ϕ *= 6%.

In fact, Figure 12 shows that *Nu*_0 _is maximum in correspondence with *H/W *= 4 for *Re *< 10000 and *H/W *= 10 for higher Reynolds numbers for all the concentrations. For *ϕ *= 0%, at *Re *= 5000 *Nu*_0 _values are about 70, 81, 86, and 87, while at *Re *= 20000, *Nu*_0 _= 195, 197, 200, and 205, for *H/W *= 4, 6, 8, and 10, respectively. The results for *ϕ *= 6% are depicted in Figure 12b; it is shown that at *Re *= 5000 the maximum value of the stagnation point Nusselt number is about 102, 100, 93, and 82, for *H/W *= 4, 6, 8, and 10, respectively. For the same geometrical configurations, at *Re *= 20000, *Nu*_0 _values are equal to 215, 225, 235, and 240.

**Figure 13:**
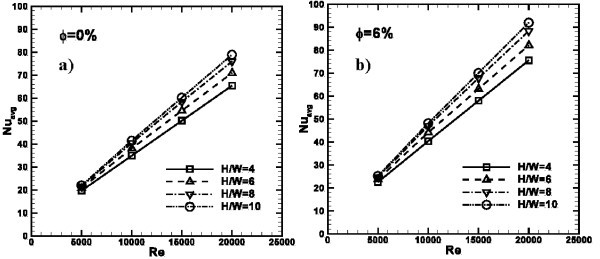
**Average Nusselt number profiles as a function of *Re *for different *H/W *ratios**: **(a) ***ϕ *= 0%; **(b) ***ϕ *= 6%.

Results in terms of average Nusselt numbers are shown in Figure 13, for different *H/W *ratios and *ϕ *= 0, 6%. The profiles increase linearly as *Re *increases as well as *H/W *ratio. In fact, the highest values of *Nu*_avg _are detected for *H/W *= 10 while the minimum ones for *H/W *= 4. Moreover, average Nusselt numbers increase as *ϕ *increases; thus, *Nu*_avg _values are equal to 42 and 79 for water, as depicted in Figure 13a, while for *ϕ *= 6%, they are equal to 48 and 92, as pointed out by Figure 12b, at *Re *= 10000 and 20000, respectively.

**Figure 14:**
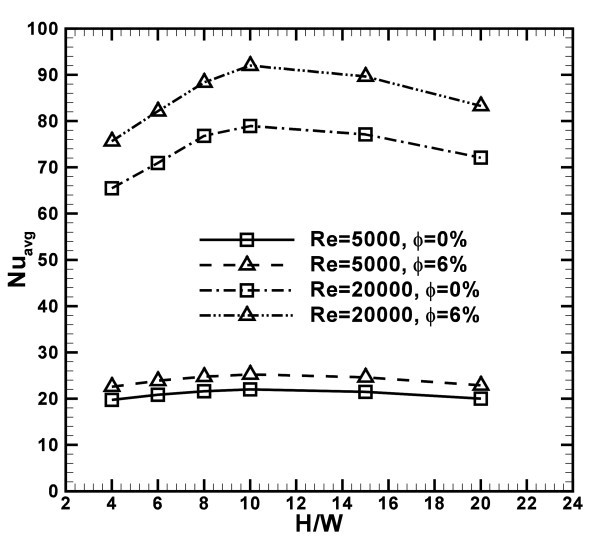
**Average Nusselt number profiles as a function of *H/W *for *Re *= 5000 and 20000, ***ϕ***= 0 and 6%**.

Figure 14 confirms that the configurations with *H/W *= 10 exhibit the maximum values of the average Nusselt numbers for all the considered Reynolds numbers and concentrations. In fact, at *Re *= 5000 and 20000, the profiles increase as *H/W *rises until *H/W *= 10, and then they decrease for *H/W *= 15 and 20.

**Figure 15:**
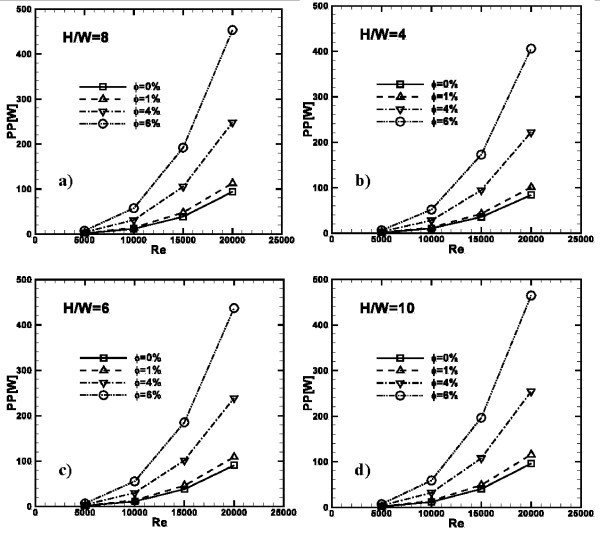
**Profiles of the required pumping power as a function of *Re*, for ***ϕ***= 0, 1, 4 and 6%**: **(a) ***H/W *= 4; **(b) ***H/W *= 6; **(c) ***H/W *= 8; **(d) ***H/W *= 10.

The pumping power is defined as PP = *V*Δ*P*, and its profiles are shown in Figure 15, for all the considered *H/W *values, concentrations and as a function of Reynolds number. The required power has a square dependence on *Re*. It increases as *H/W *and particle concentration increase. For example, as observed in Figure 15a, at *H/W *= 4, for water PP = 15 and 90 W at *Re *= 10000 and 20000, respectively, while for *ϕ *= 6% PP = 50 and 410 W. At the same *Re*, for *H/W *= 10, PP is equal to 18 W, as underlined in Figure 15d, and 98 W for water, and 58 and 470 W for *ϕ *= 6%, respectively.

**Figure 16:**
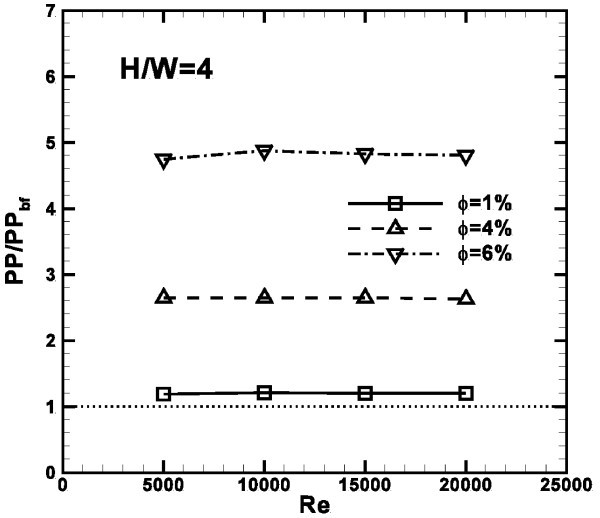
**Pumping power profile, referred to the base fluid values as a function of *Re*, ***ϕ ***= 1, 4 and 6%, *H/W *= 4**.

The pumping power ratio, referred to the base fluid values, is described in Figure 16. It is observed that the ratio does not seem to be dependent on *Re*, and PP/PP_bf _ratio increases as concentration increases, as expected. In fact, at *Re *= 15000, the required pumping power is 1.2, 2.6 and 4.8 times greater than the values calculated in case of water.

## Conclusions

A numerical analysis of a two-dimensional model on a confined impinging jet with nanofluids has been carried out to evaluate the thermal and fluid-dynamic performances and study the velocity and temperature fields. The bottom impinged wall is heated at a constant temperature and different fluid velocities are considered in the range 5000-20000. The base fluid is water and different volume concentrations of Al_2_O_3 _nanoparticles are taken into account by adopting a single-phase model approach. Furthermore, different *H/W *ratios have been studied. The dimensionless stream function contours showed that the vortex intensity and size depend on *H/W *ratio, such as on the confining effects, Reynolds number and particle concentrations. Furthermore, for increasing concentrations, nanofluids produce an increase of fluid bulk temperature, because of the elevated thermal conductivity of mixtures. The local Nusselt number profiles present the highest values at the stagnation point and the lowest at the end of the heated plate. The highest values of the average Nusselt numbers increase as the particle concentrations and Reynolds numbers increase and the highest values are observed for *H/W *= 10. A maximum increase of 18% is detected at *ϕ *= 6%. The required pumping power increases as well as Reynolds number, and particle concentrations grow, which is almost 4.8 times greater than the values calculated in the case of water. For list of symbols please see Table 4

**Table 4:** List of symbols

**Symbol**	**Quantity**	**SI Unit**
*c*_*p*_	Specific heat	J/kg K
*H*	Channel height	m
*h*	Heat transfer coefficient	W/m^2 ^K
*k*	Turbulent kinetic energy	J
*L*	Channel length	m
*Nu*	Nusselt number	Equation 11
*P*	Pressure	Pa
*PP*	Required pumping power	W
Pr = ν/a Prandtl number		
*q*	Impingement surface heat flux W/m^*2*^	
*Re*	Reynolds number	Equation 10
*T*	Temperature	K
*u*	Velocity component	m/s
$\dot{V}$	Volume flow rate	m^3^/s
*W*	Jet width	m
*x*, *y*	Spatial coordinates	m
Greek symbols		
*δ*	Kronecher delta function	
*ε*	Rate of dissipated turbulent	
	thermal energy	
*ϕ*	Nanoparticle concentration	
*λ*	Thermal conductivity	W/mK
*μ*	Dynamic viscosity	Pa s
*ρ*	Density	kg/m^3^
*σ*	Turbulent Prandtl number	
*τ*	Wall shear stress	kg/m
*ν*	Kinematic viscosity	m^2^/s
Subscripts		
0	Stagnation point	
a	Ambient	
avg	Average	
bf	Base fluid	
f	Fluid	
H	Heated	
J	Jet	
nf	Nanofluid	
p	Particle	
t	Turbulent	

## Competing interests

The authors declare that they have no competing interests.

## Authors' contributions

All the authors have made substantial contributions in order to write this work. PM and DR developed the numerical model, ran the simulation and acquired data. The analysis and the interpretation of data have been carried out together with OM and SN. All the authors have been involved in drafting the manuscript and revising it critically and OM and SN have given final approval of the version to be published.
